# Genome-wide methylome and chromatin interactome identify abnormal enhancer to be risk factor of breast cancer

**DOI:** 10.18632/oncotarget.18348

**Published:** 2017-06-02

**Authors:** Yuan Wang, Da-Peng Hao, Jing-Jing Li, Li Wang, Li-Jun Di

**Affiliations:** ^1^ Cancer Center, Faculty of Health Sciences, University of Macau, Macau, China; ^2^ Metabolomics Core, Faculty of Health Sciences, University of Macau, Macau, China

**Keywords:** enhancer methylation, breast cancer, chromatin interaction, polII ChIA-PET, gene regulation

## Abstract

Enhancer is critical cis regulatory elements in gene expression. To understand whether and how the aberrant enhancer activation may contribute to cancer risk, the differentially methylated enhancers (eDMRs) in normal and malignant breast tissues were identified and analyzed. By incorporating genome-wide chromatin interaction, integrated analysis of eDMRs and target gene expression identified 1,272 enhancer-promoter pairs. Surprisingly, two functionally distinct groups of genes were identified in these pairs, one showing better correlation to enhancer methylation (eRGs) and the other showing better correlation to promoter methylation (pRGs), and the former group is functionally enriched with cancer related genes. Moreover, enhancer methylation based clustering of breast cancer samples is capable of discriminating basal breast cancer from other subtypes. By correlating enhancer methylation status to patient survival, 345 enhancers show the impact on the disease outcome and the majority of their target genes are important regulators of cell survival pathways including known cancer related genes. Together, these results suggest reactivation of enhancers in cancer cells has the add-on effect and contributes to cancer risk in combination.

## INTRODUCTION

Gene expression is a dynamic process and is precisely regulated by many factors through mainly two layers of mechanisms including the regulation of the chromatin compaction and the regulation of the interactions between transcriptional machinery and gene regulatory elements [1–3]. Chromatin compaction status also determines if the gene regulatory elements are accessible to transcriptional factors (TFs) [4–9]. Only when the TFs can bind these gene regulatory elements, these TFs will be able to mediate the interaction between the cis-regulatory elements which may cross from tens of kilobases to as far as several megabases [10–12].

The enhancer is one kind of very important cis-regulatory elements in gene expression. A commonly recognized mechanism of the enhancer is their ability to attract the binding of activator proteins such as p300, which may be preceded by a sequence-specific factor binding and the formation of loops between the enhancer and the promoter [9, 13, 14]. Active enhancers are also enriched with unique epigenetic modifications such as the H3K4 mono-methylation and H3K27 acetylation [15]. RNA polymerase II (RNA polII), the major RNA polymerase transcribing most of the mammalian genes except the highly repetitive genes, is also an important component of enhancer binding complex [16, 17]. Although there is controversial data regarding the importance of RNA polII binding at enhancer in RNA polII recruitment to gene promoter, the enhancer does increase the pol II enrichment at the promoter and boost up the transcription activity [16, 17]. ChIA-PET, as a newly developed method, can capture the DNA fragments that contact to each other mediated by proteins [18, 19]. Pol II ChIA-PET, for instance, is able to disclose the pairs of enhancer and promoter that interact with each other mediated by PolII [20].

DNA methylation is much more stable by comparing to other histone modifications but still subjected to dynamic regulation by both methylation and demethylation mechanisms [21]. Genome-wide demethylation of DNA is frequently observed in tumors and associated with genome instability [22]. But hypermethylated tumor suppressor promoters is also very common in tumors, suggesting there are independent mechanisms to regulate the global DNA methylation versus the gene-specific DNA methylation [23]. DNA methylation contributes to gene silencing involving many closely positioned CpG sites around the gene promoter. DNA methylation occurs at these CpG sites simultaneously and creates the locally hypermethylated DNA fragment which may further recruit other factors to establish stable silencing [22, 24].

The correlation between enhancer methylation and the gene expression is rarely depicted globally owing to some technical limitations. The recent achievements in measuring DNA methylation globally and the identification of enhancers using genomic data enables the investigation of the impact of enhancer methylation on gene expression [25–28]. In this current study, we took an extra approach by incorporating the chromatin interaction ChIA-PET data, in addition to the genome-wide enhancer methylation data, in order to further narrow down the enhancers to active enhancers only in tumor samples. By examining enhancers methylation status in breast cancer patient samples, we found that gene expression has a significant correlation to their enhancer methylation, providing important evidence that aberrantly reduced enhancer methylation contributes to the differential expression of cancer-related genes, as well as the survival risk of patients.

## RESULTS

### Differentially methylated high fidelity enhancers in breast cancer

**Figure 1:**
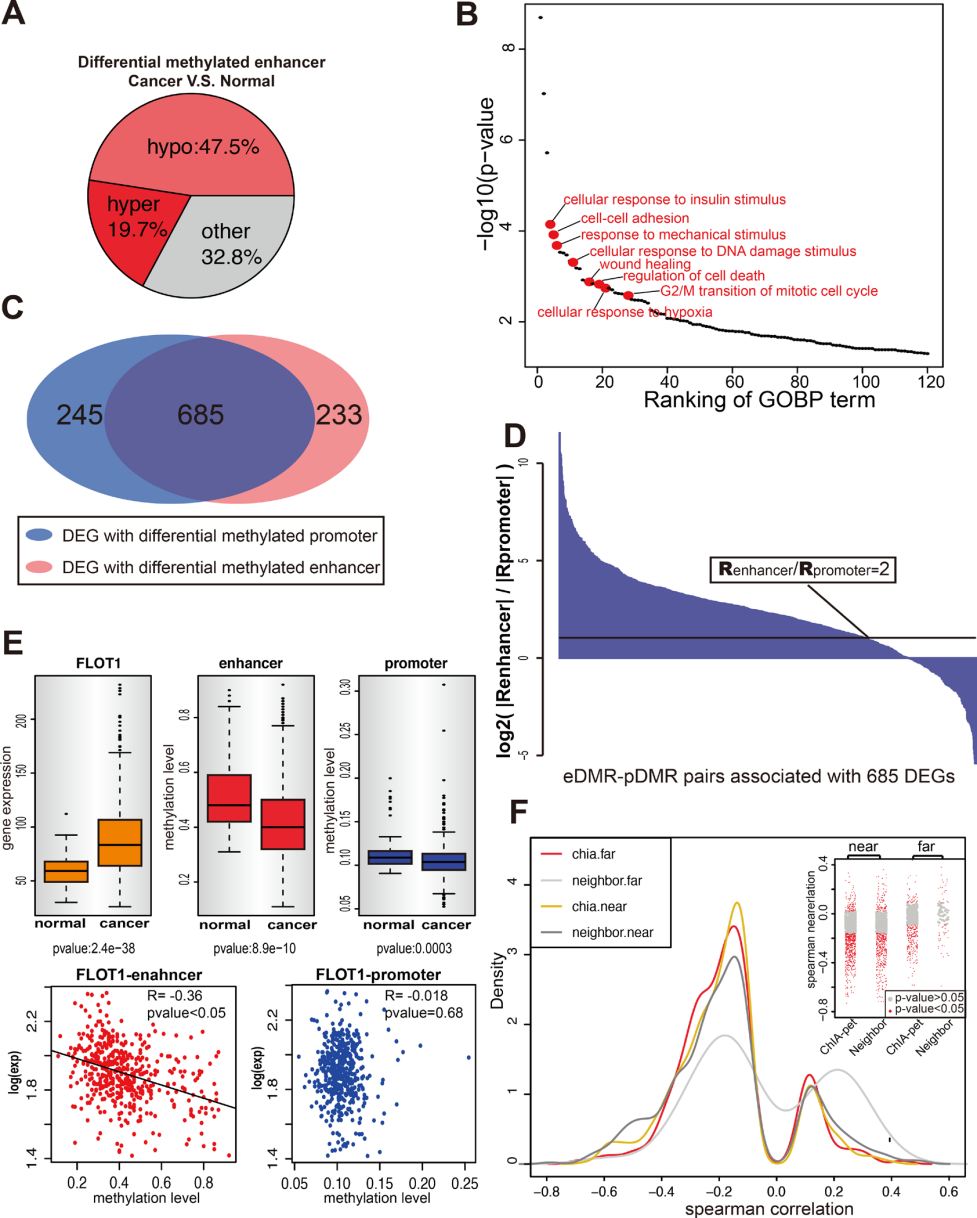
Identification of eDMRs and the target genes in breast cancer (**A**) The differentially methylated enhancers (*t*-test *p* value < 0.05) in malignant breast tissues are shown as three categories: hypermethylated, hypomethylated and others. (**B**) Gene ontology result shows eDMR target DEGs are enriched with cancer related genes. Each dot represents one GOBP term (*p* < 0.05), and the red ones are cancer-related. (**C**) Most of DEGs in 2429 E-P pairs are differential methylation for both enhancer and promoter when compare breast tumor to breast normal samples (TCGA). Venn diagram shows the number of DEGs for each part. (**D**) For the DEGs with both enhancer and promoter differential methylation, the enhancer methylation status takes the leading role of gene expression regulating. The ratio of Spearman correlation between enhancer methylation and gene expression to the Spearman correlation between promoter methylation and gene expression is indicated by the height of each bar. Each bar represents one enhancer-promoter pair. More than one pairs were counted when genes interact with multiple enhancers. The black line is the cutoff of ratio equals 2. (**E**) An example to show rather than promoter, enhancer methylation correlates with gene expression. The upper panel shows the expression of gene FLOT1 and the methylation of its enhancer and promoter in normal and cancer samples. The lower panel shows the correlation between gene expression and methylation of enhancer and promoter. (**F**) ChIA-PET is advanced in identifying remote enhancer-target pairs. Figure shows the distribution of Spearman correlation of E-P pairs identified by ChIA-PET (Red, > 100kb; Yellow, < 100kb) and E-P pairs whose promoters lied near by enhancers (Light Gray, > 100kb; Dark Gray < 100kb). The insert panel within the figure shows the Spearman correlation for each pair of each group. Every dot represents one E-P pair, and the gray region has *p*-value > 0.05.

Traditionally, enhancers are believed to be positioned to the immediate flanking regions of its regulated gene, even when one enhancer regulates multiple genes simultaneously, these genes are nearby and lineally positioned at the same locus [34–36]. Many recent studies define the enhancers using different criterions such as H3K27 acetylation, p300 binding, etc [13, 37–39]. Here we picked H3K27 acetylation to define potential active enhancers in MCF-7 cells and identified in total 14,200 active enhancers. For 3067 enhancers covered by 450K methylation array, their methylation status is compared between the normal tissues (97 samples) and tumor tissues (783 samples) from the TCGA breast cancer cohort. Totally, 68% (2062 out of 3067) of the enhancers are differentially methylated (eDMRs) between cancer and normal samples, and nearly 70% of them are hypomethylated in the tumor, which suggested enhancers tends to be active in the tumor. (Figure 1A). This result was also observed in other studies for different cancer types, such as people found 67% of eDMRs are hypomethylated in melanoma [28].

By analyzing the polII ChIA-PET data in MCF7 cells, we identified all the polII mediated chromosomal interactions firstly and obtained 2429 enhancer-promoter interaction pairs (E-P pairs) as revealed by polII ChIA-PET. Limiting by both ChIA-PET data and methylation array data, these 2429 E-P pairs are formed by 1100 enhancers and 1466 genes (Supplementary Figure 1A), of which there are 767 eDMR and 1262 DEGs (Differentially Expressed Genes between normal and tumor tissue), suggesting eDMR and DEGs are enriched in E-P pairs (Hypergeometric test *p*-value = 0.012 for eDMR and *p*-value = 4.44e–16 for DEGs). Next, the genes targeted by eDMR, non-eDMR and randomly selected genes were compared, and the DEGs are significantly enriched in eDMR group (hypergeometric distribution *p*-value: 9.8e-08) but not non-eDMR or random group (*p*-value: ~1 and 0.12), indicating DEG is more likely associated with eDMR. Functional annotation of these DEGs strongly suggests most of them are cancer-related genes (Figure 1B). Interestingly, half of these DEGs (685 out of 1262) present both enhancer and promoter differentially methylated between normal and tumor tissues (Figue 1C), suggesting the enhancer and the promoter are both required for proper regulation of these genes. To determine whether the enhancer methylation or the promoter methylation that have a greater impact on gene expression, the correlation between enhancer methylation and gene expression and the correlation between promoter methylation and gene expression were compared. As shown in Figure 1D, the ratio of these two correlations, as an indicator, suggests that enhancer is more powerful in determining the gene expression. In total, 537 of 685 genes are significantly regulated by their enhancer methylation (with *p*-value < 0.05) (Supplementary Table 1), while promoter methylation only determined 38 genes expression. The relative importance of enhancer/promoter methylation was also checked with multiple regression with relative weight analysis [64], the enhancer methylation has a consistent significant effect on gene expression (Supplementary Figure 1B). For example, FLOT1 gene, an important promoter of breast cancer cell proliferation and migration [40], shows differential expression and differential methylation of both its enhancer and promoter between normal tissues and cancer tissues. Also, the enhancer methylation correlates with FLOT1 expression much better than promoter methylation (*t*-test:*p* < 0.05) (Figure 1E), suggesting the upregulation of FLOT1 in breast cancer, as observed in many other genes as well, is dominated by the activation of its enhancer [40–44].

To compare with the traditional method which assigns enhancer to its flanking region gene, a new list of E-P pairs was established with the 3067 enhancers. As expected, the short-distance E-P pairs, whether supported by ChIA-PET or assigned by traditional method, all showed negative correlation between the methylation of enhancer and target gene expression. However, for long-distance E-P pairs (> 100kb), only E-P pairs supported by ChIA-PET showed the negative correlation (Figure 1F), suggesting ChIA-PET are much more reliable than traditional method in long-range target identification.

### Enhancer methylation directly correlates with cancer-related genes dysregulation

**Figure 2:**
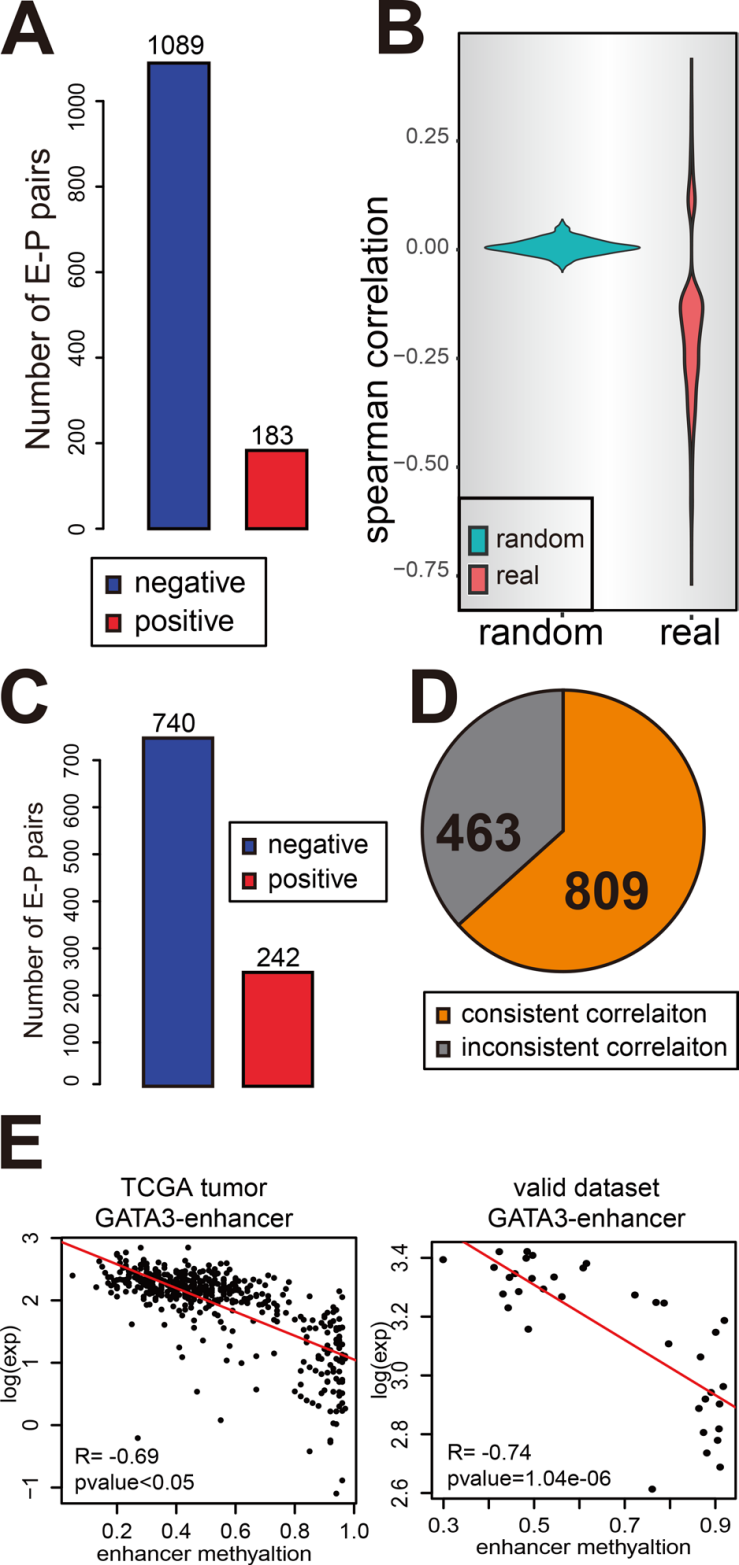
Enhancer methylation negatively correlates with gene expression (**A** and **C**) The number of negative correlated E-P pairs (blue) and positive correlated pairs (red) in TCGA breast cancer samples (A) and normal tissue samples (C). (**B**) The correlation between enhancer methylation and gene expression is not a random event. Figure shows the Spearman correlation for E-P pairs (*p*-value < 0.05) identified by ChIA-PET (red) and also random control pairs identified by randomly selected genes as target for each enhancer (green). (**D**) The result for enhancer methylation regulating gene expression can be repeated in validate dataset. The pie chart shows the portion of consistent result. (**E**) An example to show the consistent correlation in validating dataset and TCGA tumor samples.

To further validate the regulatory role of the enhancers in directing their associated gene expression, the correlation analysis was performed in the 497 TCGA breast cancer samples which have paired RNA-seq and methylation data. Of the 2429 E-P pairs identified from MCF-7 PolII ChIA-PET, 1272 E-P pairs, composed of 684 enhancers and 841 genes, present significant correlation between the enhancer methylation and gene expression (Spearman correlation *p*-value < 0.05) (Figure 2A). However, among the 23,827 DNA methylation array covered promoters, only 846 promoters show a significant correlation between the promoter methylation and gene expression. The effect of enhancer methylation on gene expression was further confirmed by comparing with the correlation between enhancer methylation and randomly selected target expression. The 684 regulator enhancers showed no correlation at all to their random targets (Figure 2B).

To further validate the correlation of enhancer methylation and gene expression, the normal breast tissues contained in TCGA dataset were also examined. Among the 1272 E-P pairs, 982 E-P pairs show a significant correlation between the enhancer methylation and gene expression (Spearman correlation *p*-value < 0.05) (Figure 2C). The same analytical procedure was applied to the validation datasets (GSE59000) which contain 36 breast cancer samples with paired gene expression and methylation data. Consistently, 809 of 1272 E-P pairs were found to have a consistent correlation between enhancer methylation and gene expression with that of TCGA tumor set (Figure 2D). Among the rest 463 inconsistent part, most of them (294) are caused by missing gene expression data as they use array instead of RNA-seq to detect expression. For example, GATA3 is strikingly high expressed in breast cancer (Supplementary Figure 2), and the correlation between GATA3 expression and its enhancer methylation are significantly negative in both TCGA tumor set and validate dataset (Figure 2E).

### Enhancer regulates gene networks

**Figure 3:**
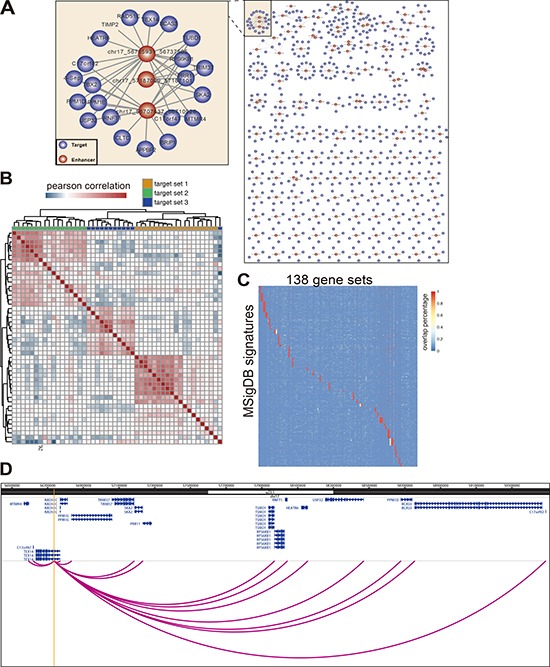
Enhancer regulates a group of functionally related genes (**A**) A network of enhancer-promoter interaction defined by polII ChIA-PET. Only show the enhancers with no less than four targets. (**B**) Genes targeted by the same enhancer tend to coexpressed with each other. Heatmap to show the Pearson Correlation of gene expression belong to 3 different enhancer target sets. Only the genes co-regulated by the same enhancer show high correlation. (**C**) Genes targeted by the same enhancer are functional related. The target genes (> 2) of each enhancer were checked for the functional correlation by mapping them to MSigDB signatures. The heatmap shows genes in the same gene set tend to lie in the same signature. The color represent the percentage of enhancer target genes overlap with signature genes. Each row represents one MSigDB signature. Each column represents one target gene set of 138 enhancers. (**D**) The genome figure shows the enhancer located in 17q22 and its interacting targets which belong to the frequently amplified genetic loci 17Q22_Q25 (one of MSigDB signature). The interactions between enhancer and target are defined by MCF-7 PolII ChIA-PET. The yellow line represents the enhancer loci.

Of noting is that one enhancer may regulate more than one promoter at the same time. For instance, one enhancer at ChrXq28 interacts with 23 promoters belonging to 17 different genes simultaneously. On the other hand, the gene YWHAZ interacts with four different enhancers simultaneously (Supplementary Figure 3A–3B). By analyzing these intricate E-P pairs, many transcriptional networks containing multiple enhancers and promoters can be obtained (Figure 3A). Degree distribution of both the enhancer and promoter in this network follows the power-law distribution, suggesting the relevance between the enhancers and their targets is functional significance (Supplementary Figure 3C).

Among the 1272 E-P pairs, there are 138 enhancers actively participating in the regulation of more than two genes at the same time (Figure 3A), and 115 of them are differentially methylated in cancer and normal samples. Importantly, the genes regulated by the same enhancer are more likely co-expressed in the tumor samples, as disclosed by the hierarchical cluster analysis of gene expression correlation when three examples of gene sets were analyzed together (Figure 3B). Additionally, more than 83.5% of the hypomethylated enhancers have their targets genes up-regulated concomitantly in breast cancer (Supplementary Figure 3D). Moreover, 119 out of 138 enhancers have their respective regulated genes in the same function term defined by MSigDB, which is very significant than random constructed E-P networks (20 out of 138), indicating that the genes regulated by the same enhancer are functionally correlated (Figure 3C). For example, the enhancer located in chr17q22 potentially regulates 16 genes, which is supported by PolII ChIA-PET data and the correlation data of enhancer methylation versus gene expression in cancer samples (Figure 3D, Supplementary Table 1). Surprisingly, all of the 16 genes are belong to the same genetic loci 17Q21_Q25, a frequently amplified chromosome region in breast cancer [45], suggesting this genetic locus is a risk factor for breast cancer.

### Divergent regulatory profile between enhancer and promoter disclosed by methylation correlated gene expression

**Figure 4:**
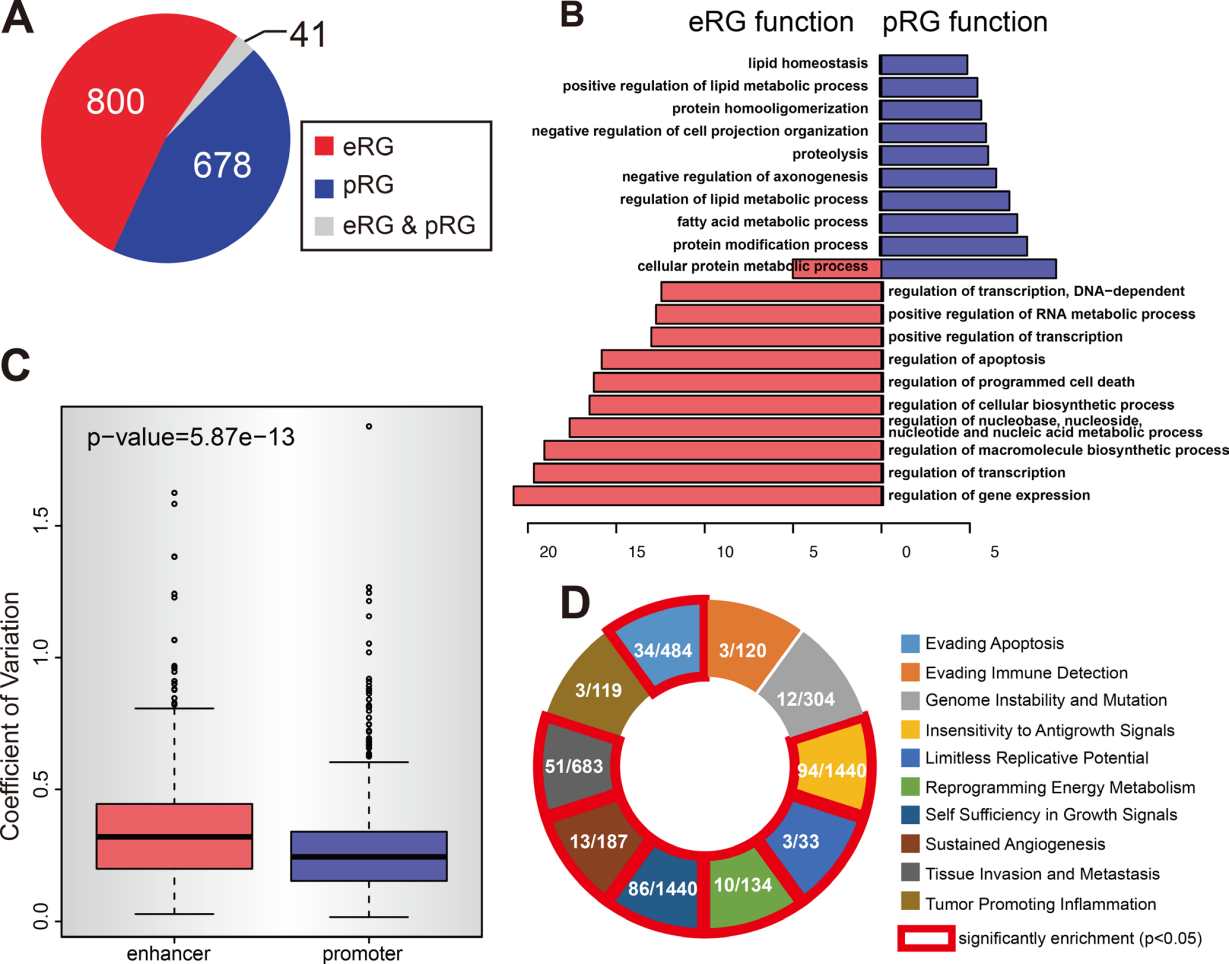
The divergent pattern of enhancer and promoter methylation in repressing gene expression (**A**) The methylation of enhancer and promoter regulate different type of genes. The pie chart shows the numbers of eRGs (enhancer regulated genes) and pRGs (promoter regulated genes). Gray part represents the genes that are both eRG and pRG. (**B**) eRGs and pRGs are different in function. Figure shows the gene ontology analysis result for eRGs and pRGs. Only the top 10 GO terms are shown for each type. (**C**) Enhancer methylation is more variable than promoter ones. Figure shows the coefficient of variation for all enhancers and promoters that are covered by 450k array. (**D**) eRGs are enriched in cancer hallmarks. Figure shows the enrichement result of eRGs for each cancer hallmark. In each box, shows the number of eRGs in hallmark (left) and the total number of genes in hallmark (right). Red line indicates significant enrichment (*p*-value < 0.05).

Previous studies suggest that promoter is enriched with CpG island and methylation of CpG island is an indicator of gene silencing [46]. But the enhancers and promoters involved in the 1272 E-P pairs show no significant preference to overlap with CpG Island (Supplementary Figure 4A). This result may be caused by the low resolution of the 450K array (Supplementary Figure 4B) because the methylation status of enhancers and promoters were calculated based solely on the CpG methylation measured by the 450K array for each sample. Correlation of gene expression and enhancer/promoter methylation identified enhancer regulated genes (eRG) and promoter regulated genes (pRG). Surprisingly, only 41 genes were identified to be both eRG and pRG, and rest of genes are either eRG or pRG (Figure 4A). This result reminds us that if there are any fundamental differences between eRG and pRG. Functional annotation identified mainly the GO terms related to metabolic activities for pRG, while eRG predominantly related to GO terms in macromolecule and RNA synthetic pathways, as well as intracellular responsiveness to stimuli including apoptosis (Figure 4B). This result indicates that pRG are more likely the housekeeping genes to maintain the static metabolism activity. eRG, however, seems to participate in the responsive activities which frequently associate with de novo RNA and protein synthesis, and sometimes the initiation of cell death programs. If this is the case, we suspect that the methylation of promoters of pRG should show less robust change among all the tumor samples comparing to the methylation of enhancers of eRG, because these pRGs should be able to maintain a steady expression even among the tumor samples. As expected, Calculation of the Coefficient of Variation (CV), a parameter to measure the variability, indicates that enhancers have significant higher CV (Figure 4C). eRG and pRG were further examined for enrichment of cancer-related pathways respectively. As expected, the eRG showed extremely significant enrichment in 7 out of 10 cancer hallmarks (Figure 4D) [47, 48]. However, pRG didn't show such result, only 1 out of 10 hallmarks was enriched by pRGs (Supplementary Figure 4C). These data suggest cancer cells are fundamentally regulated by both the exogenous factors which generate impact through the genes in response to environmental stimulations and the endogenous factors which work through the genes regulating cell death pathways.

### Breast cancer subtypes can be characterized by enhancer methylation

**Figure 5:**
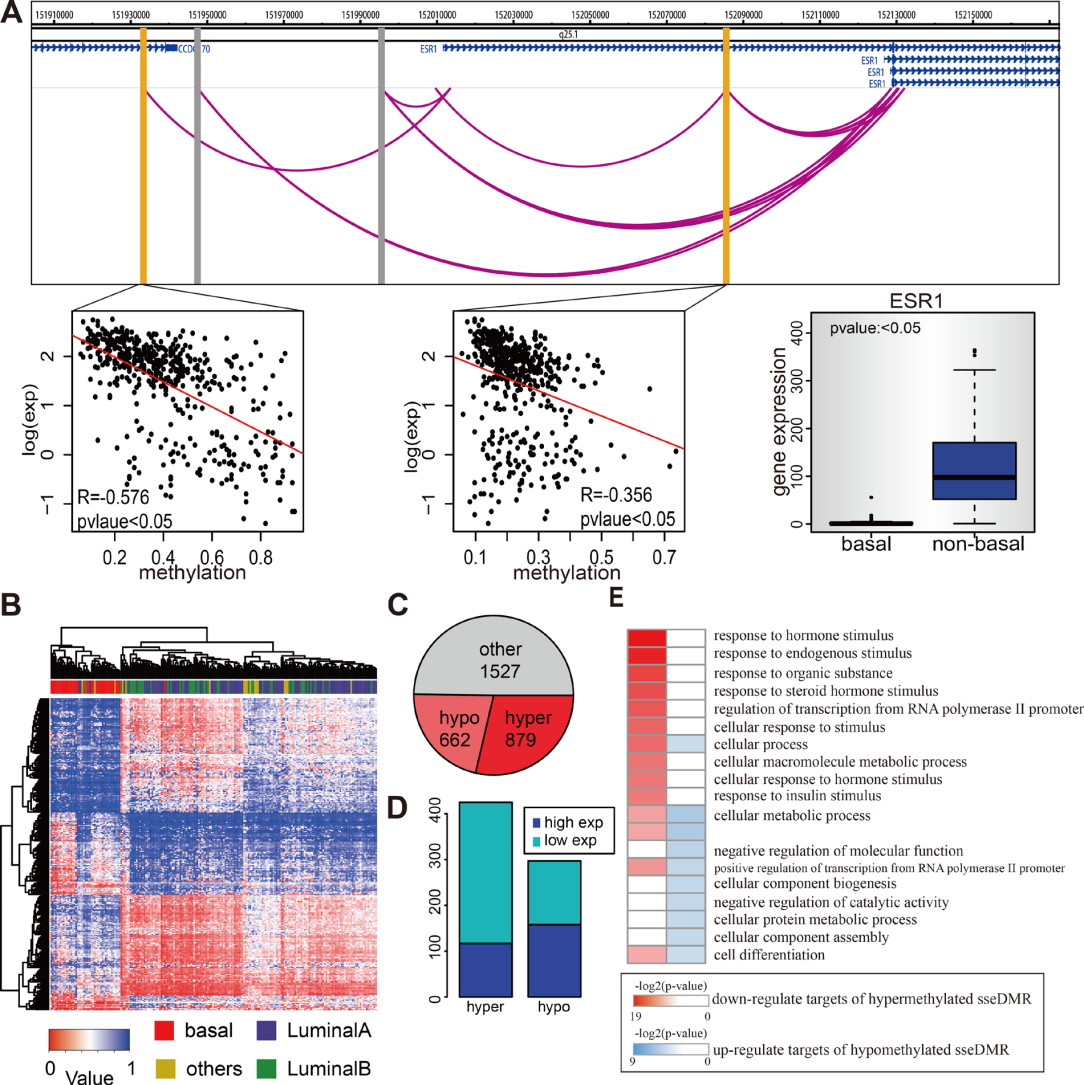
Enhancer methylation in different breast cancer subtypes (**A**) Methylation of ChIA-PET identified ESR1 enhancers are related with ESR1 expression. The genome figure shows the position of ESR1 and its four enhancers. The gray bars represent the enhancers whose methylation status is positive correlated with ESR1 expression, while the yellow bars represent the negative ones. The lower panel (from left to right) shows the correlation between enhancer methylation and ESR1 expression for those two negative correlated enhancers within TCGA tumor samples and also shows the ESR1 expression in basal and non-basal TCGA samples. (**B**) Enhancer methylation can distinguish the basal from non-basal samples. Figure shows hierarchical cluster of TCGA breast cancer dataset according to 1541 sseDMRs methylation status. Rows are the sseDMRs and columns are samples. The pathological classification of each sample is on the top. The color in the heatmap matrix is the methylation value. (**C**) The pie chart shows the number of enhancers that are hypermethylated, hypo-methylated, or no difference in basal samples comparing to non-basal samples. (**D**) The targets of hypermethylated enhancers tend to be low express in basal samples, and targets of hypomethylated enhancers tend to be high expressed. The diagram shows the number of genes for each type. (**E**) Genes actived by enhancer hypomethylation are functional different with those repressed by enhancer hypermethylation (basal V.S. non-basal). GO analysis results of these two type of genes are shown. Only top 10 go terms are shown for each type.

ESR1 gene is the coding gene of Estrogen Receptor (ER) and shows differential expression among the cancer samples, with basal-like samples the negative expression and non-basal-like the positive expression (Figure 5A), consistent with its role in distinguishing the ER-negative versus ER-positive breast cancer subtypes. Among the 1272 E-P pairs, there are four candidate enhancers, and 2 of them may directly regulate ESR1 expression, supported by significant negative correlation (Spearman *p*-value < 0.05) between the enhancer methylation and ESR1 expression. For example, the one located ~70kb upstream of ESR1 TSS has a Spearman correlation of −0.58 (*p* = 1.8e-44) (Figure 5A). Similarly, some other eRGs such as FOXA1 and ERBB2 (Supplementary Figure 5A), were also found to be differentially expressed in basal vs. non-basal breast cancer samples probably owing to the differential enhancer methylation. To explore the possibility that the enhancer methylation may be able to distinguish different breast cancer subtypes, we compared the enhancer methylation profile of basal subtype with that of non-basal subtypes and identified 1541 subtype specific enhancers (sseDMR) with differential methylation from 3067 enhancers. Importantly, clustering analysis of the enhancers identified two groups of cancer samples which matched exactly the cancer subtypes as the basal subtype and non-basal subtypes (Figure 5B). In total, 879 enhancers out of 1541 sseDMRs are hypermethylated in basal subtype, slightly more than that of 662 in non-basal subtypes (Figure 5C). Next, because we have assigned the target genes to each enhancer with PolII ChIA-PET data, the E-P pairs whose enhancers are sseDMRs can be separated into hypermethylated and hypomethylated groups according to the enhancer methylation status in basal vs. non-basal breast cancer subtypes. As expected, the hypermethylated enhancers in basal subtypes tend to have low expressed targets ( *t*-test *p*-value < 0.05 Basal V.S non-basal ), while the number of high expressed targets of hypomethylated enhancers is only slightly more than low expressed ones. (Figure 5D). To further investigate the functional significance of enhancer methylation in different breast cancer subtypes, the differentially expressed genes were used for gene ontology analysis. Interestingly, the low expressed target genes of the hypermethylated enhancers in basal subtype associated with the functions such as “response to hormones,” “response to endogenous stimulus” and response to chemicals” etc. (Figure 5E), which consistent with the features of the basal subtype of breast cancer. However, the functions enriched by all the low expressed genes in basal subtype without considering their enhancer methylation status were quite different from enhancer methylation-driven ones (Supplementary Figure 5B–5C). This observation suggests an enhancer methylation specific role in driving basal subtype associated genes dysregulation.

### Methylation status of enhancers predicts outcome of disease

**Figure 6:**
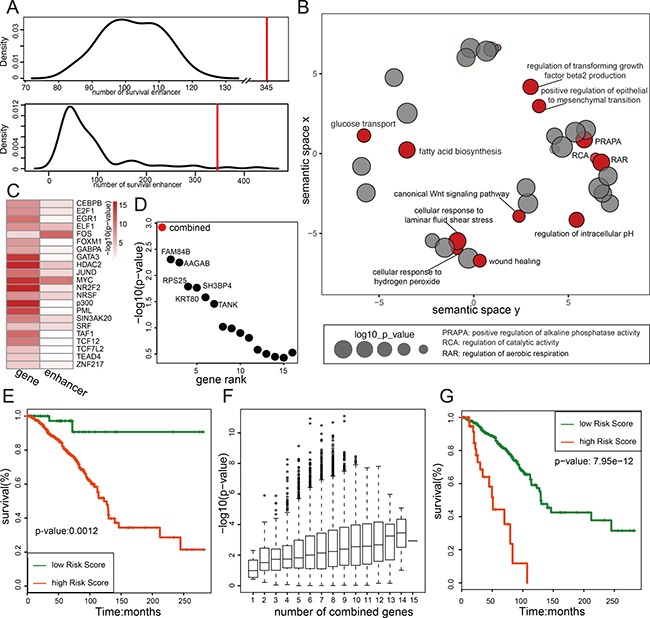
Enhancer methylation status predicts breast cancer risk (**A**) The number of survival related enhancer is larger than random. Figure shows two random analysis results, the upper panel is the number distribution of random regions whose methylation level related with patient overall survival (log-rank *p*-value < 0.05). The random region used here is selected as described in method; the lower panel is the number distribution of enhancers whose methylation level related with overall survival for perturbed patients. The red line is the number of survival enhancer in real data. (**B**) Genes regulated by methylation of survival enhancer are functional related with cancer. Go analysis result of these genes are shown in figure. The size of the node represents the enrichment *p*-value (shown in log), and red dots indicate the function associated with cancer. (**C**) The transcriptional factors enrichment result for promoters and enhancers of 120 targets of survival enhancer. (**D**) The combination of 15 breast cancer risk genes shows more effective on predicting patient overall survival. The log-ranked *p*-values of 15 breast cancer risks genes (black) and the combined *p*-values of all 15 genes (red) are shown in figure. (**E**) The overall survival curve for the combination of all 15 breast cancer risk genes. (**F**) The diversity of combination of 15 risk genes and the effect of them on prognosis. Figure shows all the log-rank *p*-value of combined survival analysis. The x-axis indicates the number of genes combined together in survival analysis. (**G**) The survival curve for the most significant combination model in survival analysis (The combination of gene CCDC83, PPFIBP2, KRT80, GADD45A, TANK, CCDC57, SLC34A1, RPS25 and TACC2).

Methylation of tumor suppressors directly correlates with the tumor initiation, progression, and metastasis. However, previous studies have mainly studied the methylation of gene promoters [49, 50]. To demonstrate the importance of enhancer methylation in breast cancer progression, we analyzed the correlation of enhancer methylation and the patient survival. Kaplan-Meier analysis of the 3067 enhancers identified 345 enhancers whose methylation status separates the patients into two distinct groups with either bad or good overall survival significantly (log-rank *p*-value < 0.05). Comparing with the random result, 345 shows a significant effect on breast cancer prognosis (Figure 6A). Within the 345 survival associated enhancers, 98 of them are potential regulators of 120 genes (also known as eRGs). Gene ontology analysis of the 120 genes identified mainly cancer related functions like “Wnt signal pathway,” “wound healing” and “positive regulation of epithelial to mesenchymal transition” (Figure 6B), suggesting these genes are important regulators of cancer progression. We also found these 120 genes and their enhancers share some transcriptional factors, such as “MYC” and “FOS,” of which the majority are transcriptional activators (Figure 6C), suggesting these transcriptional factors may mediate the active transcriptional interaction between enhancer and promoter. Within these 120 eRGs, 101 are negatively correlated with their enhancer methylation. And Cox proportional hazard regression model was used to select breast cancer risk genes from the 101 gene set. Totally 15 breast cancer risks genes were obtained (Benjamini & Hochberg FDR < 0.05) and 6 of them (FAM84B, AAGAB, RPS25, SH3BP4, KRT80, TANK) showed the most significant correlation with the patient survival (Supplementary Figure 6A). Interestingly, the combination of all those 15 risk genes showed a more significant *p*-value in correlation with patient survival than any single gene (Figure 6D–6E), suggesting an accumulative effect and each of these genes only contributes a tiny portion of the risk to the disease progression. Surprisingly, most of the enhancers of these genes (except SLC34A1) were hypomethylated, and the methylation status of them are able to distinguish the patient survival independently (Supplementary Figure 6B), suggesting an important mechanism of building up the cancer risk through turning on a group of enhancers. But we do notice that some members of this group of genes have a more significant impact on the patient risk such as CCDC83, PPFIBP2, KRT80, GADD45A, TANK, CCDC57, SLC34A1, RPS25 and TACC2, because they predict the most significant difference in patient survival when combined (Figure 6G). Among this short list, GADD45A, TANK and TACC2 are already known risk related factor in breast cancer [51–53].

## DISCUSSION

DNA methylation is represented by the methyl group modification occurring to the CpG pairs genome widely. Mechanically, the methyl group can be recognized by some proteins such as MeCPs which further bring in other repressive protein complexes including the ones building up the heterochromatin [54]. Therefore, DNA methylation frequently associates with the chromatin silencing. However, DNA methylation also goes through dynamic alteration according to the observations in recent years [21]. High-throughput DNA methylation measurement still relies on the oligo array which has very limited coverage of genome-wide CpG sites and only about 1.73% of total CpG sites are detected [55]. Some very recent studies attempt to develop other sequencing-based high-throughput technologies, but the application is still not widely used [56–58]. Also, these novel technologies can hardly be applied to analyze tumor samples. Due to the low resolution of the methylation measurement, the enhancer we studied only account for a small portion of the genome. However, functional annotation of these enhancers still confirms that methylation of these enhancers tends to control gene expression negatively and appearance of hypomethylated enhancers in cancer cells is more likely an important contributor to cancer risk. However, more analysis with genome-wide methylation data in high resolution is required to get more conclusive results.

The reactivated enhancers in cancer may reflect a truth that these enhancers are a critical responder to the micro-environment where cancer initiated, because when the microenvironment becomes unfavorable and generates the stress to the cells, the cells may choose to activate these enhancers, and therefore the downstream genes. These genes must be responsible for the responsiveness to the environmental factors. That's probably the reason that we only found very limited overlapping between the eRGs and pRGs. Moreover, the functional divergence of eRG and pRG may directly indicate the eRGs are part of the response to cancerous environment and pRG, however, responsible for sustainable cell survival.

Many studies have demonstrated the power of gene expression profiling in clustering the breast cancer samples to several subtypes and each subtype matches a particular type of cell representing a critical point in mammary gland differentiation tree [59, 60]. A known working model for enhancers to be involved in cancer is that gene fusion frequently results in the juxtaposition of oncogenes to some neighbor enhancers which activate the oncogene expression constitutively [61]. The other working model which was extensively discussed recently is the mutations occurring to enhancers which may work in cis or in trans to increase the risk of cancer [62]. But whether simply the methylation status of enhancers have an important role in regulating cancer gene expression is just the beginning to be addressed [26, 28, 63]. However, these studies only cover a very limited number of patient samples, and none of these studies focused on breast cancer. By studying breast cancer, we take advantage of the most extensive breast cancer public datasets produced by TCGA, as well as other public available breast cancer datasets. Most importantly, TCGA has the largest genome-wide methylation measurement of breast cancer patient samples, which is the only opportunity to look into how methylation of enhancers may influence gene expression in real cancer samples. As a proof of principle, we do observe the significant correlation between the enhancer methylation and gene expression. By gradually narrow down the risk enhancers, we finally obtained 15 risk genes for breast cancer and the higher than normal expression of these genes is mainly because of the hypomethylated enhancers. Thus, by increasing the resolution of enhancer methylation measurement, a novel approach to identify the risk locus of cancer is expected.

## MATERIALS AND METHODS

### Data sources

The breast cancer methylation and gene expression data are from TCGA project, which contains 777 tumor samples and 101 normal control samples for RNA-seq data and 783 cancers and 97 normals for methylation data. Within this dataset, 487 tumors and 73 normal samples have both RNA-seq and Methylation 450K Bead Array data. And the subtype and clinical information are downloaded from cBioPortal [29, 30]. The MCF-7 pol II ChIA-PET data are from two different sources, one from Encode and the other from GEO (GSE33664) with four samples. Only the high-confidence intra-chromosomal interactions supported by at least two replicates were included in this study.

The validation data used to reproduce the relationship between enhancer methylation and gene expression is from GSE59000, which contains 36 primary tumors with gene expression data and methylation data. The methylation data for MCF-7 and MDA-MB231 cell lines were downloaded from GSE65087, GSE44837 and GSE78875 [31, 32]. And all the histone binding data for MCF-7 were from Encode.

The CpG Island information and gene information were downloaded from UCSC (http://genome.ucsc.edu/) for the hg19 reference genome.

### Enhancer-target prediction

We defined the promoter region as 2kb around the gene TSS and used the H3K27ac peak of MCF-7 downloaded from Encode as potential enhancer regions. After filtering out the H3K27ac peaks that located within 2.5kB around the TSS, we used bedtools suite to determine which ChIA-PET anchors are overlapped with enhancer or promoter region and got the enhancer-target interacting pairs supported by the pol II ChIA-PET.

We used the bedtools suite to identify the closest gene as the neighbor gene for each of the enhancers we defined. To compare the correlation between enhancer methylation and ChIA-PET target gene expression to the correlation between enhancer methylation and neighbor gene expression, we filtered out the pairs whose targets are both neighbor gene and ChIA-PET target of the corresponding enhancer.

### Statistic

We used the two-side *t*-test to do the differential analysis to find eDMRs and DEGs. The correlation between methylation and expression was measured by Spearman rank correlation, and the co-expression analysis was measured by Pearson correlation. Significance was set at *p* < 0.05. We performed the gene ontology analysis with David online tools. Other enrichment analyses were done by performing the hypergeometric test. The coefficient of variation (CV) showed the extent of variability in relation to the mean, and it was calculated as follows:

$$CV=\frac{\sigma }{\mu }$$

σ, μ, represents the standard deviation and the mean.

### Risk gene identification and combination survival

We used univariate Cox regression analysis to evaluate the disease risk for each target of enhancers, and we selected the genes with FDR < 0.05 as the risk genes to do further analysis. For each gene, we could get a regression coefficient, which shows the high expression (plus) or low expression (minus) and is associated with the cancer risk.

To measure the effectiveness of the 15 breast cancer risk genes in survival prediction together, we construct a risk score with these 15 genes for each patient. The method we used to calculate the risk score is the same with a published paper [33]. Briefly, the formula considered both the direction and the power of genes in cancer risk and also took the expression into account. The risk score for each patient can be calculated as follows:

$$Risk_{score}=\sum_{i=1}^{15}\beta_{i}*Exp_{gene\left(i\right)}$$

β_*i*_ is the regression coefficient for gene i in previous analysis and the Exp is the expression of gene i in that patient. We split the patients with overall survival time into two group according to if the risk score is larger than 0 or not, and working with Kaplan-Meier analysis to check if the risk score can use as a predictor to predict the patient survival outcome.

### Random analysis

We random selected the genes from the total 20502 genes detected by RNA-seq as enhancer target and calculated the correlation between the enhancer methylation and gene expression. We compared the random result with the real ones to show the regulation effect of enhancer on gene expression.

To strengthen the enhancer methylation effect in breast cancer prognosis, we create a simulated ‘enhancer’ set by randomly selected the CpG site and extended it by adding 500bp length to each side to construct a fragment. The total number of enhancers we used in this analysis is 3067, so we random constructed 3067 fragments to do survival analysis. We did this random process for 100 times and compared the random results to the real one. Besides, we also did samples perturbation for 100 times to further support our result.

### random E-P networks

To show the genes regulated by the same enhancer are functionally correlated, we randomly constructed 138 E-P subnetworks. For each subnetwork, we randomly selected the genes from the total gene pool as the targets of respective enhancer and the number of the genes is arbitrarily from 2 to 16, which is the smallest and largest target number of the real subnetwork for 138 enhancers.

## SUPPLEMENTARY MATERIALS FIGURES AND TABLE





## Abbreviations

eDMR: differential methylation enhancer
pDMR: differential methylation promoter
eRG: enhancer methylation regulated gene
pRG: promoter methylation regulated gene
DEG: differential expressed gene
sseDMR: subtype specific differential methylation enhancer
E-P pair: enhancer-promoter pair
