# Supplementary material for: Genome-wide methylome and chromatin interactome identify abnormal enhancer to be risk factor of breast cancer
Source: Oncotarget. 2017 Jun 2;8(27):44705–19. doi: 10.18632/oncotarget.18348 (PMC5546512; doi:10.18632/oncotarget.18348)
Supplement: Supplementary file 1 [file oncotarget-08-44705-s001.pdf]

Genome-wide methylome and chromatin interactome identify abnormal enhancer to be risk factor of breast cancer

Supplementary Materials

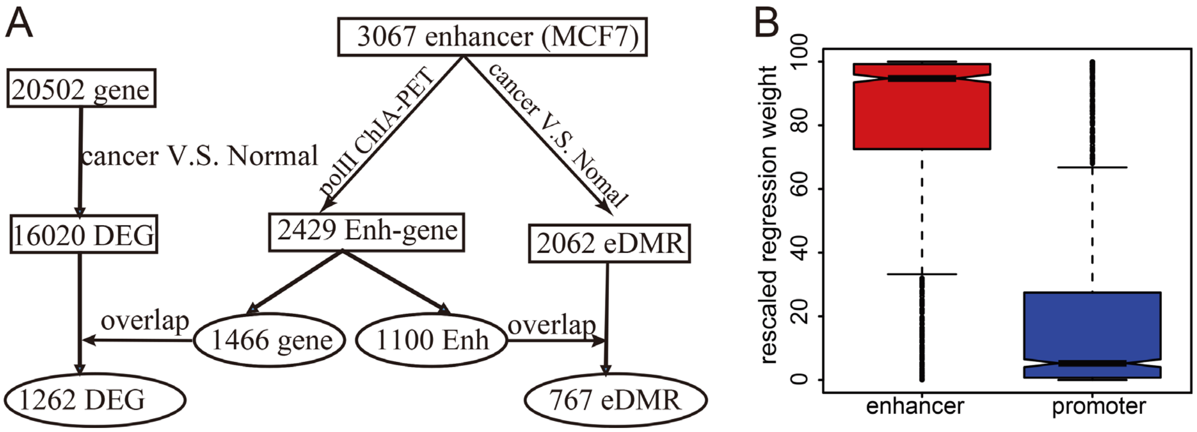

Supplementary Figure 1: (**A**) The pipeline of data processing in identifying ChIA-PET supported differential expressed genes (DEG) and differentially methylated enhancers (eDMR). (**B**) Enhancer methylation takes the leading role in gene expression regulating. The boxplot shows the weights of the enhancer (red) and promoter (blue) in multiple regression with relative weight analysis.

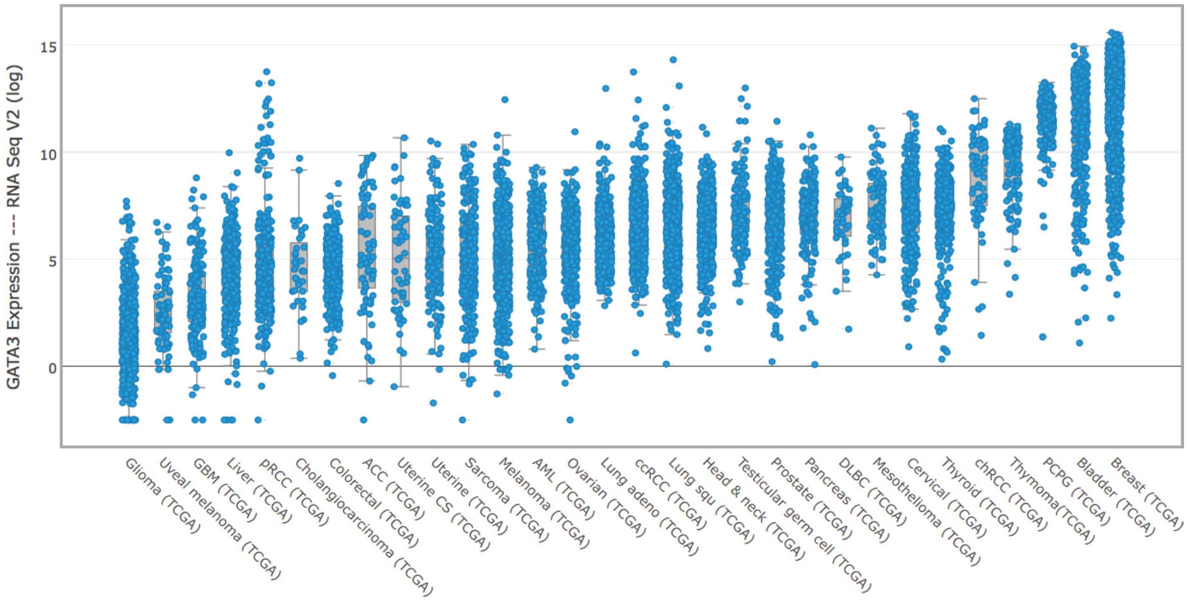

* Figure from cBioPortal

Supplementary Figure 2: The expression of GATA3 in other cancer types of TCGA dataset. The figure comes from cBioPortal online results.

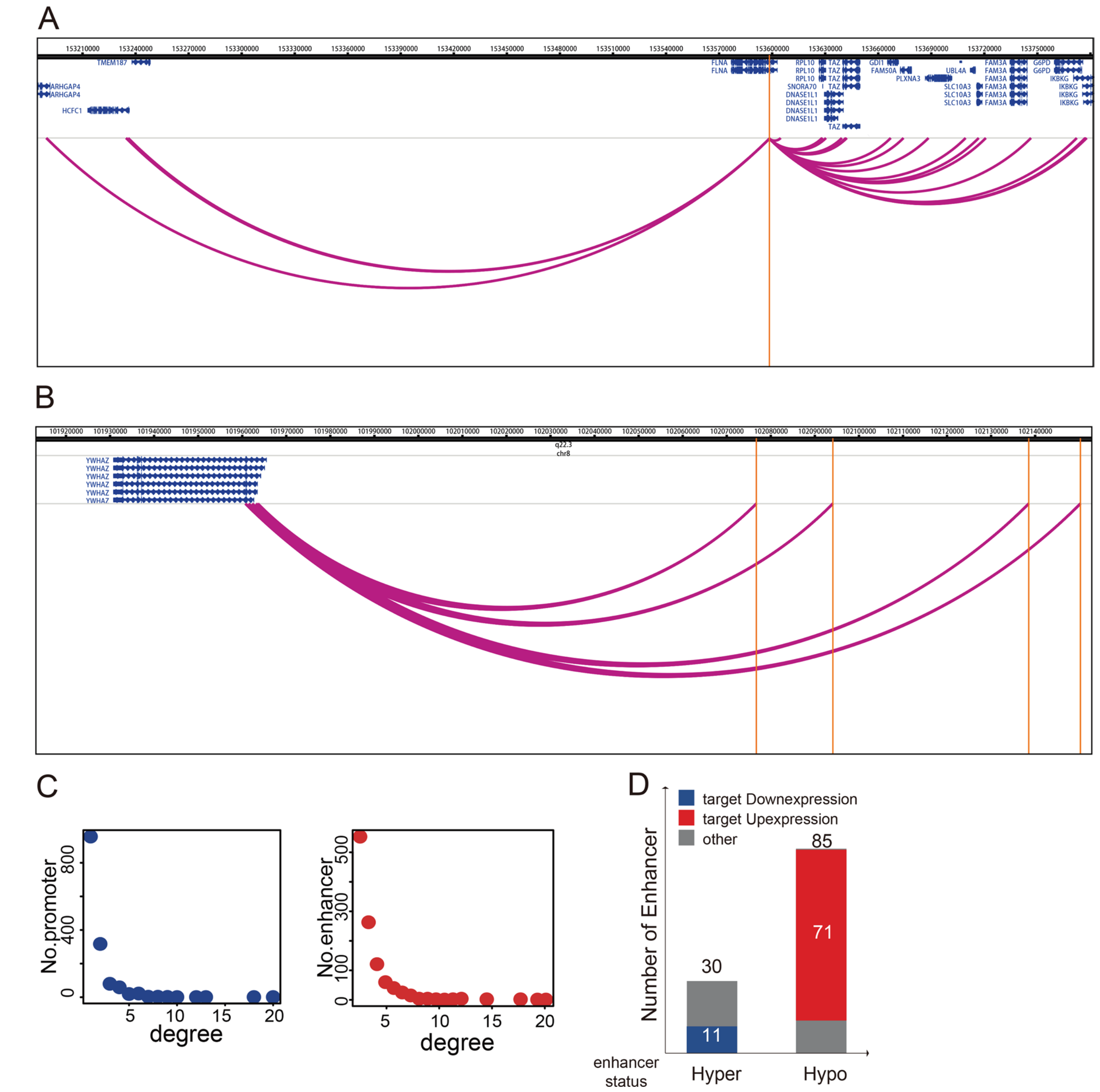

Supplementary Figure 3: (**A**) An example to show one enhancer can interact with multiple genes. and (**B**) is an example to show one gene can be targeted by more than one enhancer. The yellow lines represent enhancer loci. All the interactions are defined by MCF-7 PolII ChIA- PET. (**C**) The degree distribution of enhancers and genes in the network construction. (**D**) Hypomethylated enhancer accounts for high gene expression. The diagram shows the number of hypermethylated enhancers with their target genes downregulated; hypomethylated enhancers with their target genes upregulated.

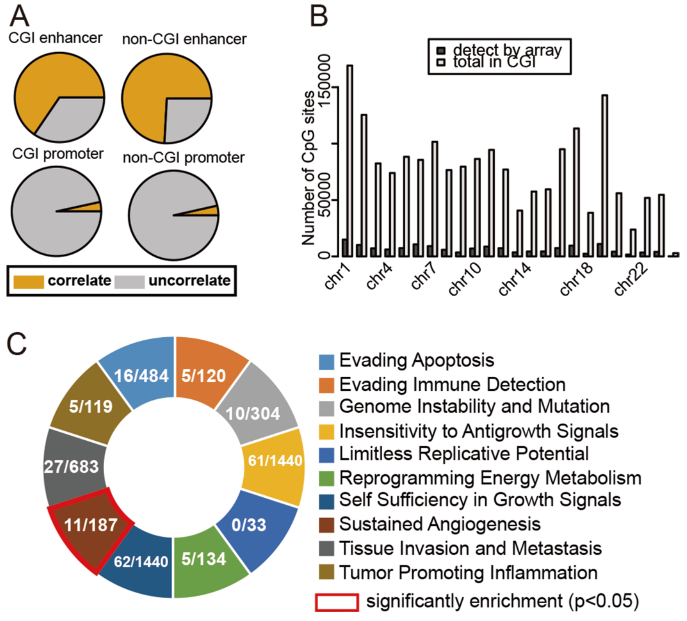

Supplementary Figure 4: (**A**) The number of enhancers/promoters whose methylation correlated with gene expression is not obviously different between two group: enhancer/promoter overlap with CGI and enhancer/promoter without CGI. (**B**) The resolution of methylation 450K array is not sufficient. The number of CpG sites contained in every CGI (gray) and the number of CpG sites detected by the 450k array in every CGI (red). (**C**) The enrichment of pRGs (promoter methylation regulated genes) in 10 cancer hallmarks (red indicates significant enrichment (*p*-value < 0.05)).

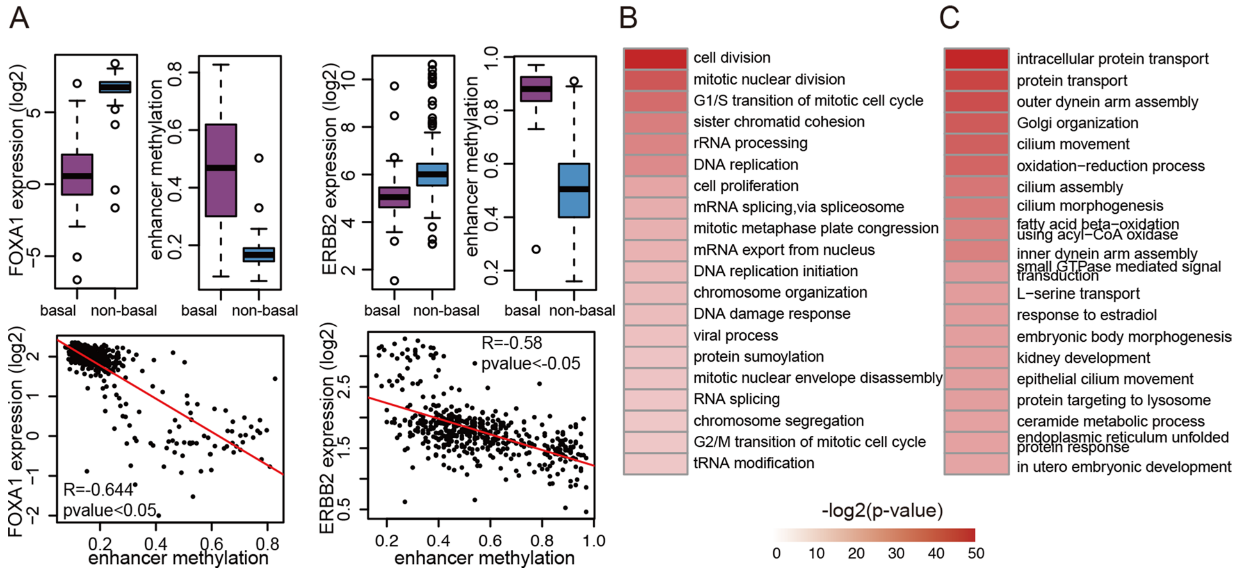

Supplementary Figure 5: (**A**) Other example to show breast cancer subtype associated genes may be regulated by enhancer methylation. Up panel shows the gene expression of FOXA1 and ERBB2 and methylation level of their enhancers in basal and non-basal TCGA samples. Lower panel shows the correlation between enhancer methylation and gene expression. (**B**) The top 20 GOBP terms enriched by all genes high expressed in the basal subtype. (**C**) The top 20 GOBP terms enriched by all genes low expressed in the basal subtype.

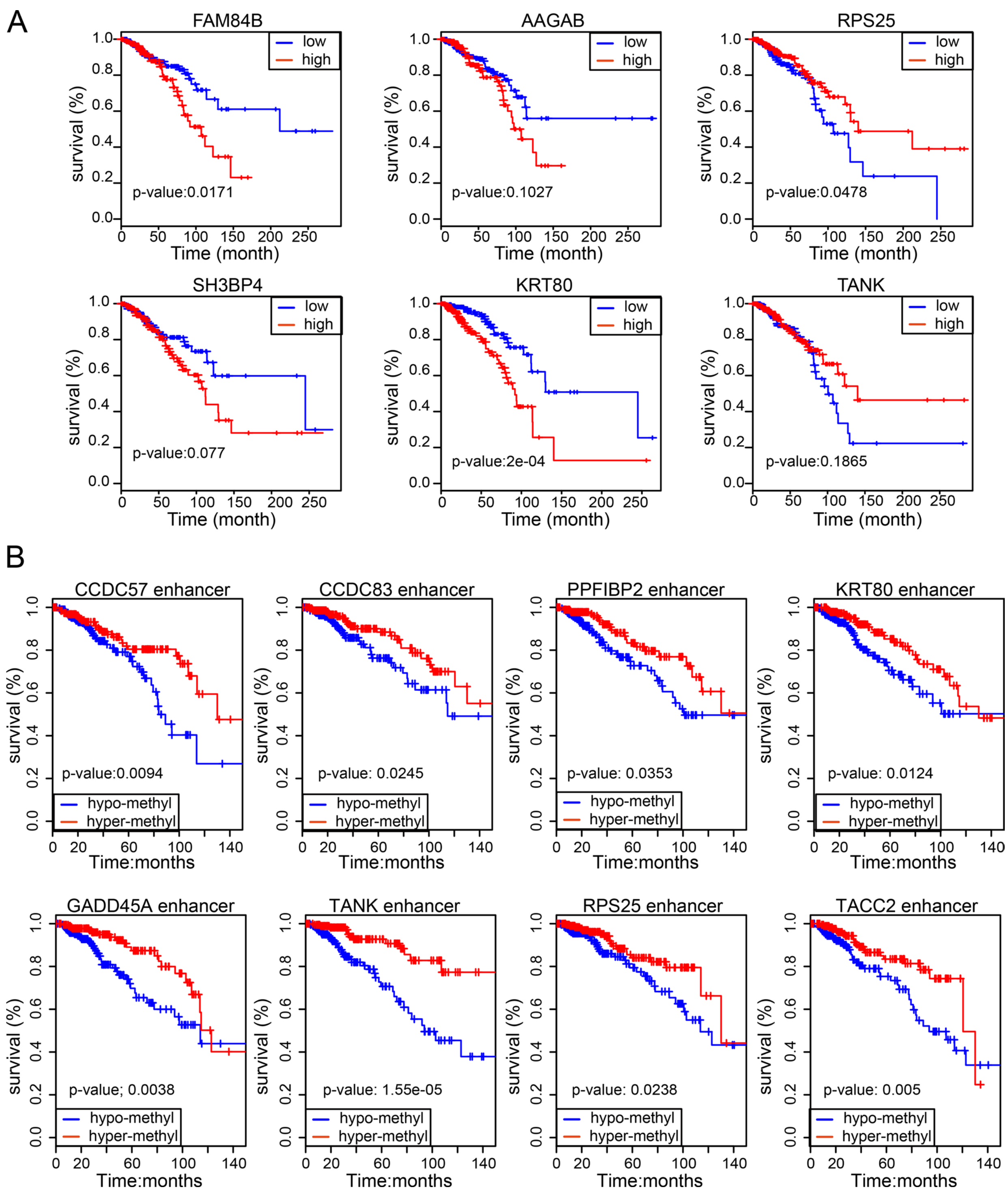

Supplementary Figure 6: (**A**) Survival curves of the most six survival related genes selected from 15 breast cancer risk genes. (**B**) The survival curves of the enhancers of 15 selected genes (except SLC34A1 enhancer who shows hypermethylation in breast cancer.)

Supplementary Table 1: The correlation for all 2429 Enhancer-gene pairs supported by MCF7 PolII ChIA-PET are listed. See_Supplementary_Table_1
